# Expression patterns of ethylene biosynthesis genes from bananas during fruit ripening and in relationship with finger drop

**DOI:** 10.1093/aobpla/pls041

**Published:** 2012-12-24

**Authors:** Olivier Hubert, Didier Mbéguié-A-Mbéguié

**Affiliations:** 1 CIRAD, UMR QUALISUD, F-97130 Capesterre-Belle-Eau, Guadeloupe, France; 2 CIRAD, UMR QUALISUD, F-34398 Montpellier, France

## Abstract

**Background and aims:**

Banana finger drop is defined as dislodgement of individual fruits from the hand at the pedicel rupture area. For some banana varieties, this is a major feature of the ripening process, in addition to ethylene production and sugar metabolism. The few studies devoted to assessing the physiological and molecular basis of this process revealed (i) the similarity between this process and softening, (ii) the early onset of related molecular events, between the first and fourth day after ripening induction, and (iii) the putative involvement of ethylene as a regulatory factor. This study was conducted with the aim of identifying, through a candidate gene approach, a quality-related marker that could be used as a tool in breeding programmes. Here we examined the relationship between ripening ethylene biosynthesis (EB) and finger drop in order to gain further insight into the upstream regulatory steps of the banana finger drop process and to identify putative related candidate genes.

**Methods:**

Postharvest ripening of green banana fruit was induced by acetylene treatment and fruit taken at 1–4 days after ripening induction, and total RNA extracted from the median area [control zone (CZ)] and the pedicel rupture area [drop zone (DZ)] of peel tissue. Then the expression patterns of EB genes (*MaACO1*, *MaACO2*, *MaACS1*, *MaACS2*, *MaACS3* and *MaACS4*) were comparatively examined in CZ and DZ via real-time quantitative polymerase chain reaction.

**Principal results:**

Differential expression of EB gene was observed in CZ and DZ during the postharvest period examined in this study. *MaACO1*, *MaACS2* and *MaACS1* were more highly induced in DZ than in the control, while a slight induction of the *MaACS4* gene was observed. No marked differences between the two zones were observed for the *MaACO2* gene.

**Conclusions:**

The finger drop process enhanced EB gene expression including developmental- and ripening-induced genes (*MaACO1*), specific ripening-induced genes (*MaACS1*) and wound-induced genes (*MaACS2*). Thus, this process might be associated with a specific ethylene production in DZ of the pedicel area and the result of crosstalk between developmental, ripening and wound regulatory pathways. *MaACO1*, *MaACS1*, *MaACS2*, and to a lesser extent *MaACS4* genes, which are more highly induced in DZ than in CZ, could be considered as putative candidates of the finger drop process.

## Abstract (precis)

Ethylene biosynthesis genes were transcriptionnally enhanced by finger drop during banana fruit ripening. Among them, there are ethylene- and ripening- (*MaACO1, MaACS1*) and wounded-related (*MaACS2*). Thus, this process might be associated with ethylene production and share in common some components with wounding, another breaking event.

## Introduction

Ethylene is a gaseous plant hormone that regulates many developmental events and biotic and abiotic stress responses of plants. Banana fruits undergo a climacteric ripening process. This is characterized by a ripening-related increase in respiration and a burst of ethylene production concomitantly with physicochemical and biochemical changes, including chlorophyll breakdown, increased starch degradation and sugar synthesis, and fruit softening.

Finger drop is a key feature that is closely associated with ripening of some banana varieties. This phenomenon has a substantial economic impact for the banana marketing sector. Indeed, bananas are marketed in hands of generally 4–9 fruits. Dislodgement of individual fruits from the hand at the pedicel area considerably reduces the commercial value of the product because hands with missing fingers or fingers without pedicels cannot be sold to consumers. Despite this economic importance, very few studies have been devoted to this phenomenon.

The finger drop process was first observed in bananas of the Cavendish subgroup in 1934 (Hicks 1934). It was defined as physiological softening and weakening, thus causing individual fruit in a hand to separate from the crown (Baldry *et al.* 1981). The sensitivity to finger drop within *Musa* germplasm varies according to the variety and ploidy (New and Marriott 1983; Pereira *et al*. 2004), growing and postharvest ripening, and storage conditions (Semple and Thompson 1988; Paull 1996; Saengpook *et al*. 2007). At the biochemical level, changes in water-soluble pectin, i.e. a cell wall polysaccharide component, have also been reported to be associated with finger drop. In addition to the activities of some cell wall hydrolases including pectate lyase and polygalacturonase, an increase in water-soluble pectin has been observed in the drop zone (DZ) as compared with control fruit (Imsabai *et al.* 2006). Recent molecular studies performed in Cavendish bananas also showed that a change in the expression of major cell-wall-modifying genes occurs specifically in the finger drop area (Mbéguié-A-Mbéguié *et al*. 2009). Overall, major molecular changes in the expression of genes coding for cell-wall-modifying proteins (CWMPs) occurred 1–4 days after ripening induction, but in a sequential manner. Firstly, there were changes in the expression of pectolytic and cell-wall-loosening genes, mainly during Days 1–2, followed by changes in the expression of xyloglucan genes, mainly during Days 3–4. The fact that some CWMP genes are involved in both finger drop and the fruit-softening process, with transcriptional regulation by ethylene, suggests that these two processes that occur during banana fruit ripening might involve common regulatory mechanisms and factors, with ethylene being one of them.

Numerous physicochemical, biochemical and molecular findings have shown that fruit softening is one of the ripening physiological processes that is most sensitive to ethylene (Gerasopoulos and Richardson 1996; Jiang *et al*. 1999; Kim *et al*. 1999; ; Flores *et al*. 2001; Grimplet 2004; Hayama *et al*. 2006; Johnston *et al*. 2009).

In higher plants, including banana, 1-aminocyclopropane-1-carboxylic acid (ACC) synthase (ACS) and ACC oxidase (ACO) catalysed the two key steps of the ethylene biosynthesis (EB) pathway: the formation of methionine via *S*-adenosyl-l-methionine (AdoMet) and the cyclic non-protein amino acid ACC, respectively. In banana, ethylene production displays a sharp peak at the onset of ripening, followed by a rapid decrease thereafter due to a slight decline in the ACC content and a sharp decrease of *in vivo* ACO activity attributed to the availability of its cofactors, especially iron and ascorbate (Liu *et al*. 1999; Choudhury *et al*. 2008).

The ACS and ACO proteins have been widely studied at both biochemical and molecular levels in different species. ACC oxidase and ACS are encoded by a multigenic family whose members are differentially regulated at transcriptional and translational levels by various developmental environmental and hormonal signals (Bleecker and Kende 2000; Wang *et al*. 2002; Lin *et al*. 2009). At least nine ACS and three ACO genes have been isolated from banana and published in the literature or directly registered in the GenBank database (Liu *et al*. 1999; Huang *et al.* 2006; Inaba *et al.* 2007). However, only a few of them were fully characterized in regard to ripening and none in regard to the finger drop process. The ACS and ACO genes are transcriptionally and differentially regulated according to the tissue and stimuli. One ACS (*MaACS1*) and two ACO (*MaACO1* and *MaACO2*) genes were found to be ripening related (Liu *et al*. 1999; Huang *et al.* 2006; Inaba *et al.* 2007). Post-translational regulation has also been reported in several plants, and mainly for ACS enzymes. This regulation is based on the C-terminal region of the ACS protein that can be directly phosphorylated by protein kinase, leading to an increase in protein stability (Lin *et al*. 2009; Han *et al*. 2010; Kamiyoshihara *et al*. 2010; Skottke *et al*. 2011; Wang *et al*. 2011; Choudhury *et al*. 2012), or bound to a substrate-specific adaptor, i.e. the ETO1 protein, and directed to the ubiquitin (Ub)/26S proteasome system (Chae and Kieber 2005; Yoshida *et al*. 2005). The ACS activity enzyme encoded by a ripening-related gene *MaACS1* from banana was also subjected to post-translational regulation during fruit ripening. A few studies also reported a proteolytic cleavage of the protein leading to a decrease in the apparent pI of the protein and its inactivation, suggesting that this protein might also be under post-translational regulation as was ACS protein (Barlow *et al*. 1997; Ramassamy *et al*. 1997).

In this study, we investigated, at the transcriptional level, the putative relationship between the finger drop process and EB, i.e. two processes that occur during banana fruit ripening. To this end, the transcript abundance of ACS and ACO genes from banana was estimated comparatively in the median zone and DZ during the ripening of bananas harvested at the commercial maturity stage. Our findings suggest a possible role of ethylene and ripening-regulated elements in the regulation of the finger drop mechanism.

## Methods

### Fruit harvesting and ripening induction

The banana fruits (*Musa acuminata*, AAA, Cavendish, cv Grande Naine) used in this study were collected from at least four banana plants taken randomly from a banana farm near the CIRAD research station (elevation 250 m; andosol; rainfall 3500 mm/year), Guadeloupe (French West Indies). Banana plants were grown under conventional field practices and, on the basis of heat concept unit (Umber *et al*. 2011), fruits were harvested at commercial maturity stage [i.e. 900 degree-day (dd)], which corresponds to ∼90 days after flowering.

After harvest, internal fruit of the median hand of all banana bunches, considered to be comparable (Liu 1976), were pooled and kept for 24 h at 20 °C in chambers. Fruits were then placed into sealed Plexiglas boxes, and their ripening was induced by injection of 1000 p.p.m. acetylene and the boxes were kept for 24 h at 20 °C and ambient humidity. At the end of treatment, fruits were removed from the boxes and kept ripening at 20 °C in air and ambient temperature. During postharvest ripening, a sample of three fruits was taken daily to assess the postharvest ripening process and finger drop development.

### Assessment of the physiological stage of fruit and measurement of finger drop development during postharvest ripening

The physiological stage of fruit was assessed through measurement of soluble solid content (SSC). To this end, 5 g of fresh powder of pulp tissue were homogenized in an equivalent volume of distilled water and the mixture was centrifuged for 10 min at 10 000 × *g* and 4 °C. The fruit juice was collected and the SSC was determined using a digital Refracto 30PX/GS refractometer from Mettler Toledo (Grosseron, Saint-Herblain, France) and expressed in Brix. The development of finger drop was measured as previously described (Chillet *et al*. 2008).

All experiments were performed on three fruits at each time point. Immediately after these analyses, peel tissue corresponding to the median part of the fruit [control zone (CZ)] and to the rupture area of the fruit pedicel (DZ), and pulp tissue were sampled separately, frozen in liquid nitrogen and stored at −80 °C until use for total RNA extraction and gene expression analysis.

### RNA extraction and quantitative real-time polymerase chain reaction analysis

Total RNAs were extracted twice from a pooled sample tissue using a modified hot borate method (Wan and Wilkins 1994; Mbéguié-A-Mbéguié *et al*. 2008*b*). At each developmental stage, peel tissue from three fruits was pooled due to the small quantity of peel material obtained per fruit, mainly from the DZ, thus making it difficult to blend in a coffee grinder.

The relative expression of each transcript was determined in triplicate on two independent RNA extracts by quantitative real-time polymerase chain reaction (qPCR) using a 7500 Real-Time PCR System (Applied Biosystems, Courtaboeuf, France). The first-strand cDNA synthesis was performed from 2 µg of RQ1-DNase-treated RNA from each RNA extract using a random hexamer primer and *MMLV* reverse transcriptase (Promega, Charbonnières, France) according to the manufacturer's instructions. The synthesized cDNA was diluted 1 : 10 with distilled water, and 5 μL of the diluted cDNA and gene-specific primer were used as the template for qPCR analysis in a 20 µL volume reaction, as previously described in Mbéguié-A-Mbéguié *et al*. (2008*a*). All primer sequences used in this study are listed in Table 1.

**Table 1:** **Sequences of gene-specific primers used in this study**. The actin, *MaACO1*, *MaACO2* and *MaACS2* gene-specific primers used in this study were those previously described by Mbéguié-A-Mbéguié *et al*. (2008*a*). *MaACS1*, *MaACS3* and *MaACS4* primers were designed using primer-BLAST software (Rozen and Skaletsky 2000) and mainly within the 3′-untranslated region for *MaACS1*, and within the coding region for *MaACS3* and *MaACS4* sequences (Liu *et al*. 1999; Inaba *et al.* 2007). Each assay using the gene-specific primers amplified a single product of the correct size, and the PCR efficiency (slope) of primers within the 86–99 % range (3.7–3.37) was calculated as described in Mbéguié-A-Mbéguié *et al*. (2008*a*)

Gene	Primer name	Sequences	Annealing temperature	Product size (bp)
*MaACT*	Act-F	GAGAAGATACAGTGTCTGGA	60	231
	Act-R	ATTACCATCGAAATATTAAAAG		
*MaACO1*	ACO1-F	AAGCTCTACGTCGGGCATAA	60	152
	ACO1-R	GACAGCTTCCTAACGCGAAG		
*MaACO2*	ACO2-F	CCAAGGAACCGAGATTTGAA	60	125
	ACO2-R	TGGTAGCTTCCACGATGACA		
*MaACS1*	ACS1-F	AGAACTCCTCCTACTTCGAT	60	215
	ACS1-R	ATGATAGTCCTGAAAGTTGG		
*MaACS2*	ACS2-F1	TGCGGCCTTGTTCTGCTGGG	60	151
	ACS2-R1	AAACCACCCCGGTTCGTCGC		
*MaACS3*	ACS3-F1	CCGTACTATCCAGGGTTCGACAGGG	60	231
	ACS3-R1	GAAGTCGACGAGGGTGTCCAGTTCT		
*MaACS4*	ACS4-F1	GCAGAAGCGTGGCCTCAGGG	60	166
	ACS4-R1	CGAGTCGAAGCTGGTGCCCG		

The relative fold differences in expression of each gene between samples were determined using the 2^−ΔΔCt^ formula (Livak and Schmittgen 2001) with the actin gene as reference and the fruit CZ of peel tissue sampled at harvest before ripening induction used as calibrator.

## Results

### Finger drop pattern during postharvest fruit ripening

**Fig. 1:**
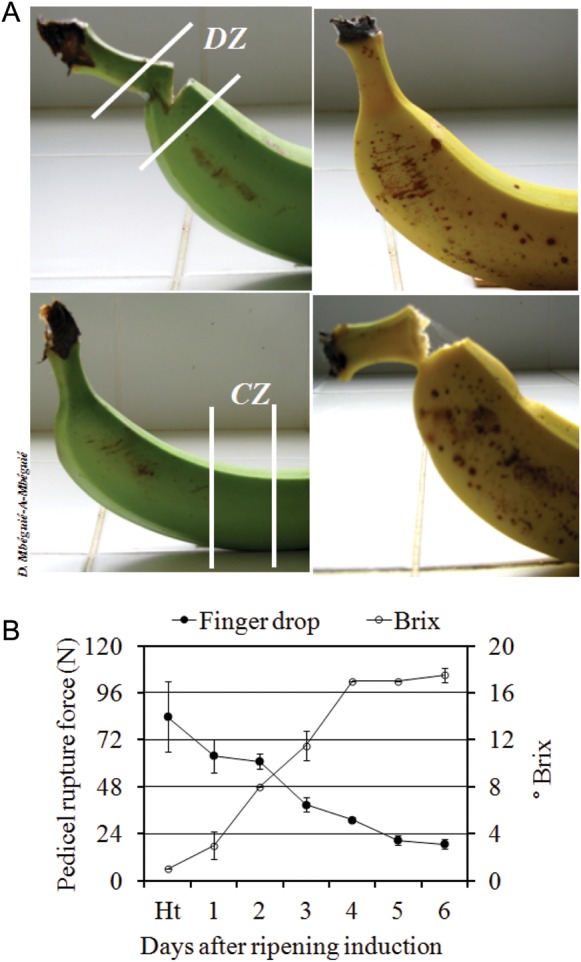
**Finger drop pattern during postharvest fruit ripening**. Peel tissue of control (CZ) and drop (DZ) zones used in this study are illustrated in (A). Finger drop pattern observed during fruit ripening and measured via the pedicel rupture force are illustrated in (B).

During postharvest ripening of acetylene-treated banana fruit, the SSC content estimated via the Brix value started to increase at Day 1 after ripening induction and increased progressively until Day 4, when it reached its maximum. The Brix value remained constant from Day 4 to Day 6 (Fig. 1). According to the rupture force measurement, Cavendish banana finger drop started 1 day after ripening induction and continued progressively throughout the postharvest ripening stage. However, a marked decrease was observed between Days 1 and 3 after ripening induction, with a more than 2-fold decrease in the pedicel rupture force. As the pedicel rupture force pattern is considered to be an effective way of measuring banana finger drop (Saengpook *et al*. 2007), our data suggest that our experimental conditions induced the development of the finger drop process in Cavendish bananas.

### Expression of EB genes in peel tissue from CZ and DZ during banana fruit ripening

**Fig. 2:**
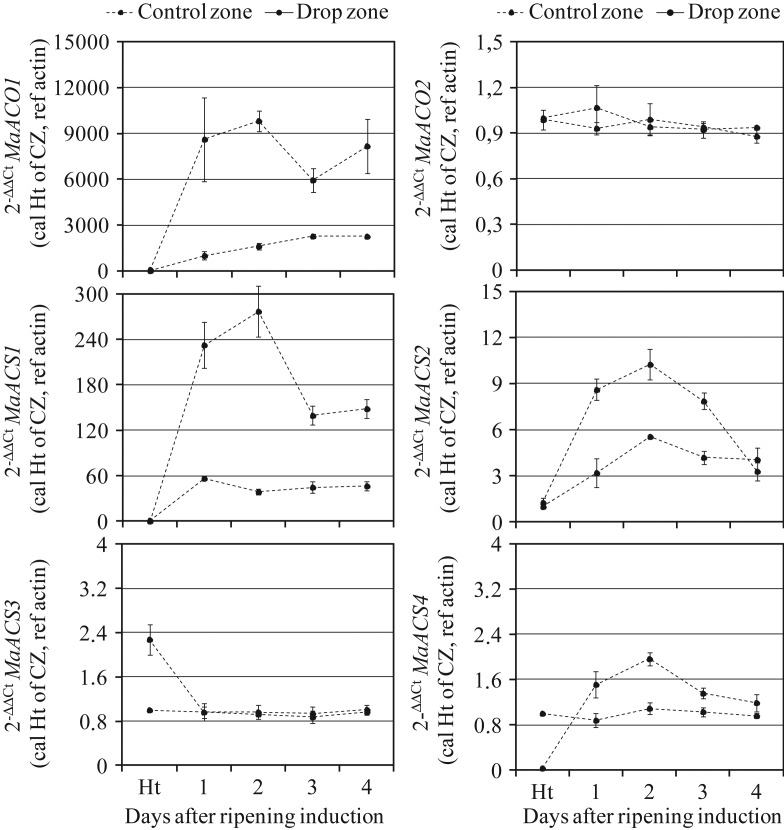
**Ethylene biosynthesis gene expression during postharvest ripening of Cavendish bananas assessed using reverse transcription–polymerase chain reaction**. RNA was extracted twice from CZ and DZ of banana peel tissues sampled at harvest and then at 1–4 days after ripening induction. The *y*-axis represents the relative fold difference in mRNA level and was calculated using the 2^−ΔΔCt^ formula (Livak and Schmittgen 2001) with actin as the reference. The mRNA fold difference was relative to that of peel tissue from the CZ of fruit sampled at harvest. Each data point is the mean of values obtained through a qPCR reaction performed in triplicate on one sample. Each sample was prepared from four fruits originating from three replicate bunches. Vertical bars indicate the standard deviation (SD). The SD was lower than the symbol when no bar is shown. The experiment was performed on two independent RNA extractions with similar results.

The expression profiles of six EB genes, including two ACO and four ACS, were studied during postharvest ripening of banana fruit harvested at the commercial mature green stage and treated with acetylene (Fig. 2).

No change was observed in the *MaACO2* mRNA level in both CZ and DZ tissues. At harvest, the *MaACS3* level was 2-fold higher in the DZ compared with the control. This level decreased markedly at Day 1 after ripening induction to reach a level comparable to that observed in the CZ, and then the *MaACS3* mRNA level remained constant in both tissues. The other EB genes, i.e. *MaACO1*, *MaACS1*, *MaACS2* and *MaACS4*, were highly and transiently induced in DZ compared with the control, *MaACO1* and *MaACS4* being the most and least expressed ones, respectively. For the *MaACS1*, *MaACS2* and *MaACS4* genes, mRNA accumulation peaked 2 days after ripening induction. *MaACS4* was the unique gene presenting, at harvest time, a low transcript level in the DZ compared with the control.

## Discussion

Finger drop is a major fruit ripening feature for some banana varieties. Our findings confirmed this by showing a progressive decrease in rupture pedicel force, concomitantly with finger drop sensitivity, throughout fruit ripening, along with a decrease in the SSC, as expressed by the Brix value (Fig. 1). Our previous findings also showed that molecular mechanisms related to the banana finger drop phenomenon occurred earlier after ripening induction as was ripening ethylene production (Mbéguié-A-Mbéguié *et al*. 2009). This led us to suggest ripening ethylene as a potential upstream regulator of the finger drop process. Assuming that this putative regulation—if it takes place—might involve EB components, in this study we examined the changes in expression of EB genes during postharvest ripening of banana fruit taken at 1–4 days after ripening induction, and in both median zone (CZ) and DZ.

Ethylene biosynthesis genes are transcriptionally induced in peel tissue by ripening (i.e. an increase in their mRNA accumulation in the median zone), *MaACS1* and *MaACO1* being the highly expressed ones, consistent with the important role played by these genes in ripening EB (Liu *et al*. 1999; Inaba *et al.* 2007), while no marked changes were observed in the expression of *MaACS3*, *MaACS4* and *MaACO2* genes in CZ, suggesting that these genes might be less important during ripening of banana peel tissue. In contrast with previous studies showing that *MaACS2* gene was expressed only in banana pulp tissue and upon wounding (Liu *et al.* 1999), our data showed a low but transient induction of *MaACS2* in control zone of peel suggesting that this gene was ripening regulated in peel tissue. The discrepancy between the two data may be due to the analytical method, i.e. northern blot used by Liu *et al.* (1999) and qPCR used in this study.

The finger drop process enhanced EB gene expression, including the MaACO1, MaACS1, MaACS2 and MaACS4 genes, of which the corresponding mRNA was accumulated to a great extent in DZ compared to the control. Therefore, we hypothesized that the finger drop process is associated with ethylene production occurring specifically in the DZ with a putative involvement of one ACC oxidase (*MaACO1*) and three ACC synthase (*MaACS1*, *MaACS2* and *MaACS4*) genes. However, this hypothesis needs to be validated through the measurement of ethylene produced by peel tissue taken from DZ in comparison to that taken from the median zone.

The *MaACO1* and *MaACS2* genes previously identified as wound-inducible genes in pulp and leaf banana tissues (Liu *et al*. 1999; Mbéguié-A-Mbéguié *et al*. 2008*a*) were also transcriptionally enhanced by finger drop. Although the tissues and physiological stages examined in these previous studies are different, our data suggested that finger drop might also imply a wound-related mechanism. Considering that ripening and wounding processes are both associated with ethylene production, it should be interesting to assess whether finger drop, wounding and ripening share some ethylene transduction pathway components.

**Fig. 3:**
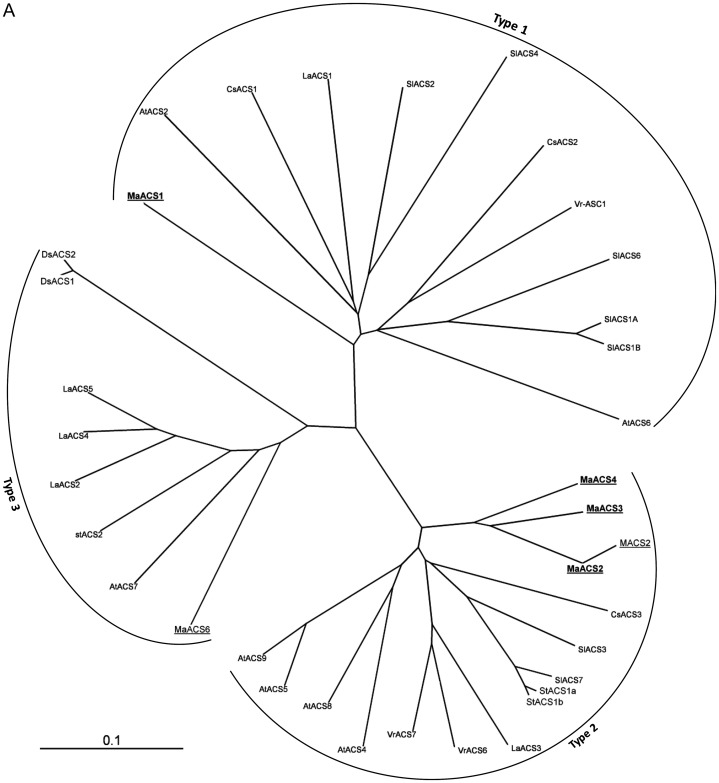

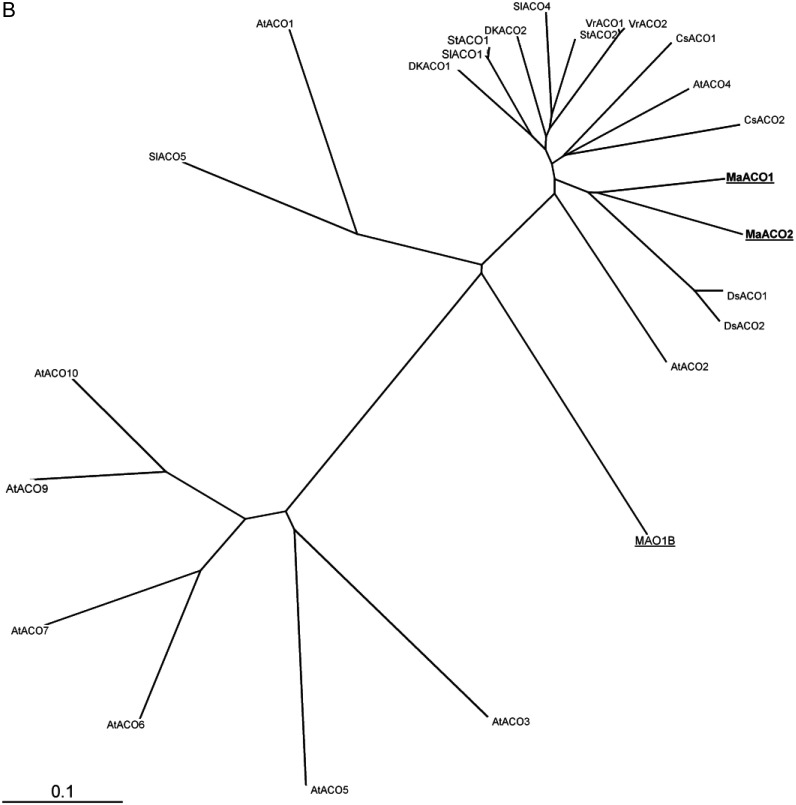
**Phylogenetic analysis of ACS (A) and ACO (B) sequences from banana and other plant species.** The phylogenetic tree was constructed with the Gonnet residue weights and after a complete sequence alignment performed using the ClustalX algorithm (Jeanmougin *et al*. 1998). For multiple alignments, the gap opening penalty was 10, with a gap extension penalty of 0.2 and the delay divergent sequences was set at 30 %. The consensus tree was displayed using the TREEVIEW program (Page 1996). All banana sequences are indicated by underlining while those whose expression was examined in this study are in bold. ACC synthase sequences used for phylogenetic tree construction are: *Musa acuminata* [*MaACS1* (AB021906), *MaACS2* (AB021907), *MACS2* (AF056162), *MaACS3* (AB021908), *MaACS4* (AB266314), *MaACS6* (AJ223186)], *Arabidopsis thaliana* [*AtACS2* (Q06402), *AtACS4* (NP_179866), *AtACS5* (AAG50098), *AtACS6* (Q9SAR0), *AtACS7* (AAG48754), *AtACS8* (AAG50090), *AtACS9* (AAG48755)], *Cucumus sativum* [*CsACS1* (BAA33374), *CsACS2* (BAA33375), *CsACS3* (BAA33376)], *Doritaenopsi* sp*.* [*DsACS1a* (L07882), *DsACS1b* (L07883)], *Lupinus albus* [L*uACS1* (AF119411), *LuACS2* (AF119412), *LuACS3* (AF119413), *LuACS4* (AF119410), *LuACS5* (AF119414)], *Solanum lycopersicon* [*SlACS1a* (AAF97614), *SlACS1b* (AAF97615), *SlACS2*(CAA41855), *SlACS3*(AAB48945), *SlACS4*(AAA03164), *SlACS6*(BAA34923), *SlACS7*(AAC32317)], *Solanum tuberosum* [*StACS1a* (Z27233), *StACS1b* (Z27234), *StACS2* (Z27235)] and *Vigna radiata* [*VrASC1* (CAA77688), *VrACS6* (U34986), *VrACS7* (U34987)]. ACC oxidase sequences used for phylogenetic tree construction are: *Musa acuminata* [*MaACO1*(AY804252), *MaACO2* (X95599), *MAO1B*(AF030410)], *Arabidopsis thaliana* [*AtACO1* (AEC06898), *AtACO2* (AEE33960), *AtACS3* (Q8H1S4), *AtACS5* (Q43383), *AtACS6* (AAG48754), *AtACS7* (AAG50090), *AtACS9* (AAG48755), *AtACO10* (Q9LSW6)], *Cucumus sativum* [*CsACO1* (AB006806), *CsACO2* (AB006807)], *Diospyros kaki* [*DkACO1* (AB073008), *DkACO2* (AB073009)], *Doritaenopsis* sp*.* [*DsACO1* (L37103), *DsACO2*(L07912)], *Solanum lycopersicon* [*SlACO1* (X58273), *SlACO4* (AB013101), *SlACO5* (AJ715790)], *Solanum tuberosum* [*StACO1* (AF384820), *StACO2* (AF384821)] and *Vigna radiata* [*VrASO1* (U06046), *VrACO2*(AM180697)].

ACS and ACO genes are encoded by a large and small multigenic family, respectively. In contrast to ACO, the members of which are highly conserved, the ACS multigenic family was classified into three types according to the consensus motifs present at their C-terminal polypeptide (Yoshida *et al*. 2005). It has been stated that individual members of the ACS and ACO multigenic families were not restricted to only one function. In order to assess the relationships between the structure of ACS and ACO polypeptides and their putative function, an unrooted phylogenetic tree was constructed from a multiple alignment of ACS and ACO polypeptides registered in the database (Fig. 3). Three major lineages can be discerned from the ACS phylogenetic tree (Fig. 3A). *MaACS1* belongs to Type 1 ACC synthase. Indeed, the *MaACS1* C-terminal region presents the main features of Type 1 ACS protein including the Ser residues in the ‘RLSF’ motif, necessary for CDPK phosphorylation (Chae and Kieber 2005), followed by a 27-amino-acid tail containing the two Ser residues 476 and 479 recently proved to be phosphorylated during banana fruit ripening (Choudhury *et al*. 2010, 2012), and finally the absence of the ‘WVF’ binding ETO1 motif (Yoshida *et al*. 2005), which is degenerated to ‘WDEAL’. *MaACS2*, *MaACS3* and *MaACS4* polypeptide sequences are grouped in the same cluster, which is clearly divergent from *MaACS1*.

A previous study suggested that MaACS2, MaACS3 and MaACS4 are members of the same subgroup of the ACS family (Liu *et al*. 1999; Inaba *et al.* 2007). Consistent with this, the phylogenetic tree includes *MaACS2*, *MaACS3* and *MaACS4* into a divergent subgroup family of Type 2 ACS. However, this needs to be confirmed with a phylogenetic tree constructed with the complete *MaACS2*, *MaACS3* and *MaACS4* polypeptide sequences, as those used here are partial and lack the last 80 amino acid residues. Two main subfamilies are observed for the ACO phylogenetic tree (Fig. 3B). Banana ACO genes examined in this study are grouped with the other major ACO genes, consistent with the high conservation of these proteins. Based on the present data (i.e. the limited number of ACO and ACS genes examined), a putative relationship between ACO and ACS lineages and their corresponding function in regard to finger drop cannot yet be established. A more detailed analysis of the expression of the members of both ACO and ACS family genes is necessary before assigning a functional homology to this lineage. This is now possible with the availability of the banana genome sequence (D'Hont *et al*. 2012).

## Conclusions and forward look

In conclusion, our data showed that the finger drop process might be associated with ethylene production, implying a large number of EB genes. However, this hypothesis needs to be validated through the measurement of ethylene produced at DZ.

The finger drop process is probably a result of the crosstalk between ethylene (i.e. ripening and wounding) and developmental regulatory pathways. The ethylene transduction pathway model has been proposed and the related components identified (Cara and Giovannoni 2008). On the other hand, MADS-box genes have recently been identified as a major component in the molecular circuit of the developmental regulation of fruit (Vrebalov *et al.* 2002; Giovannoni 2004; Elitzur *et al.* 2010). It should be interesting to identify the ethylene and developmental transduction components involved in the finger drop process. This represents an interesting challenge to gain further insight into the banana ripening process and especially the physiological events occurring in banana peel tissue, whose ripening process clearly differs compared with that of pulp.

Finally, with the prospect of identifying ripening-related markers through a candidate gene approach, *MaACO1*, *MaACS1*, *MaACS2*, and to a lesser extent *MaACS4* genes that are induced in DZ to a greater extent than in CZ could be considered as putative candidates related to the upstream regulatory steps of the finger drop process. However, and before their use in molecular breeding schemes for banana improvement, these candidates need to be validated through functional studies using cultivars contrasting their tendency to develop finger drop.

## Sources of funding

The work is a part of the ongoing research project ‘Qualité des Produits Végétaux’ funded by the Fonds Européen de Dévéloppement Régional (FEDER) Convergence: Guadeloupe 2007–2013.

## Contributions by the authors

D.M.-A-M. designed the project, performed all the molecular biology experiments, constructed the phylogenetic tree, analysed the qPCR data (Fig. 2) and wrote the manuscript. O.H. performed all the physicochemical experiments, including fruit sampling, treatment, monitoring of postharvest ripening and physicochemical data analysis described in Fig. 1.

## Conflict of interest statement

None declared.
